# POCUS Diagnosis of Bacterial Lymphadenitis Later Determined to be Cat Scratch Disease: A Unique Presentation and Review

**DOI:** 10.24908/pocusj.v10i02.19399

**Published:** 2025-11-17

**Authors:** Alec P. Tolentino, Stanley Wojtas, Camille D. Audette, Erin J. Meyer, Zachary W. Binder

**Affiliations:** 1 UMass Chan Medical School, Worcester, MA, USA Chan Medical; 2 Department of Pediatrics, UMass Chan Medical School, UMass Memorial Children's Medical Center, Worcester, MA, USA

**Keywords:** POCUS, Inguinal Lymphadenopathy, Bartonella, Cat Scratch Disease

## Abstract

Acute lymphadenopathy has a wide range of possible etiologies, ranging from self-limiting viral infections to life-threatening malignancy. Point of care ultrasound (POCUS) can play a crucial role in identifying lymphadenopathy and ruling out other potential causes of soft tissue swelling, such as hernias, abscesses, and malignancies. Furthermore, POCUS enables the characterization of lymphadenopathy by evaluating the size, shape, echogenicity, and vascularity of the involved lymph nodes, thereby helping to identify the presence and extent of pathology. When performed at the time of initial presentation, POCUS can narrow the differential diagnosis and guide appropriate work-up and management. We present a case of a teenage male evaluated in the pediatric emergency department for acute bilateral inguinal swelling, in which POCUS identified lymphadenopathy and guided the evaluation of a presumed infectious process, ultimately determined to be caused by cat scratch disease.

## Introduction

The differential diagnosis for soft tissue swelling is broad and includes entities such as hernias, abscesses, neoplasms, and lymphadenopathy. Point of care ultrasound (POCUS) can be used in the initial evaluation of soft tissue swelling. Specific POCUS findings, such as the size and shape of the structure, presence of critical features (bowel with or without peristalsis, a hilum, free fluid, surrounding edema or cobblestoning, posterior acoustic enhancement), and flow patterns on color Doppler, can aid in determining the diagnosis (see Tables 1 and 2) [1–4].

**Table 1.** Causes of soft tissue swelling and their point of care ultrasound (POCUS) characteristics [1,2].

Cause of Soft Tissue Swelling	POCUS Findings
Non-Incarcerated Hernia	Peristalsing bowel Changes size with Valsalva
Incarcerated Hernia	Non-reducible Absent peristalsis Bowel wall thickening; Free fluid
Lymphadenopathy	Well defined; Ovoid No change with Valsalva See Table 2 for further characterization
Abscess	Heterogenous +/− internal debris Adjacent soft tissue edema common Adjacent cobblestoning common
Neoplasm	Solid Non-compressible Variable echogenicity Variable vascularity

**Table 2.** Point of care ultrasound (POCUS) features of various lymph node pathologies [3,4].

POCUS Features	Normal	Reactive	Infectious/Suppurative	Lymphoma
Shape	Ovoid	Ovoid	Ovoid	Round
Visible Hilum	+	+	+/−	−
Cystic Necrosis	−	−	+/−	−
Posterior Acoustic Enhancement	−	−	+	−
Adjacent Soft Tissue Edema	−	−	+	−
Color Doppler	Hilum	Hilum	Peripheral “Fire Pattern”	Mixed

Lymphadenopathy is a common cause of soft tissue swelling. While most often reactive, benign, and self-limited, lymphadenopathy can be a symptom of a more severe localized or systemic disease, such as infection or malignancy, and requires further investigation [5]. In cases of inguinal lymphadenopathy, potential etiologies include herpes simplex, lymphogranuloma venereum (LGV), chancroid, lymphoma, syphilis, lower extremity skin infections, and penile or urethral carcinomas [6].

When lymphadenopathy is identified, POCUS is particularly valuable in helping to delineate the underlying etiology by assessing for specific sonographic features [3]. We present a case in which POCUS, performed immediately after physical examination and before any further workup, guided the evaluation and management of acute bilateral inguinal swelling in the pediatric emergency department. We then describe the POCUS findings in the context of the patient's ultimate diagnosis, cat scratch disease (CSD).

## Case Report

A 16-year-old boy presented to the pediatric emergency department for painful bilateral inguinal swelling. He first developed pain and swelling in the bilateral inguinal regions ten days prior to presentation. Over the three days immediately preceding his presentation, the right inguinal swelling doubled in size with the development of overlying erythema. The patient had no significant past medical or surgical history, took no medications regularly, and had no known allergies. He lived with multiple pets, including a dog, two cats, and a turtle. He did not recall any recent animal bites or scratches. He was sexually active with one female partner and had no prior history of sexually transmitted infections. He regularly shaved his pubic hair with a razor and had cut himself while shaving a few days before symptom onset. His review of systems was positive for night sweats, decreased appetite, and difficulty falling asleep due to pain.

**Figure 1.**
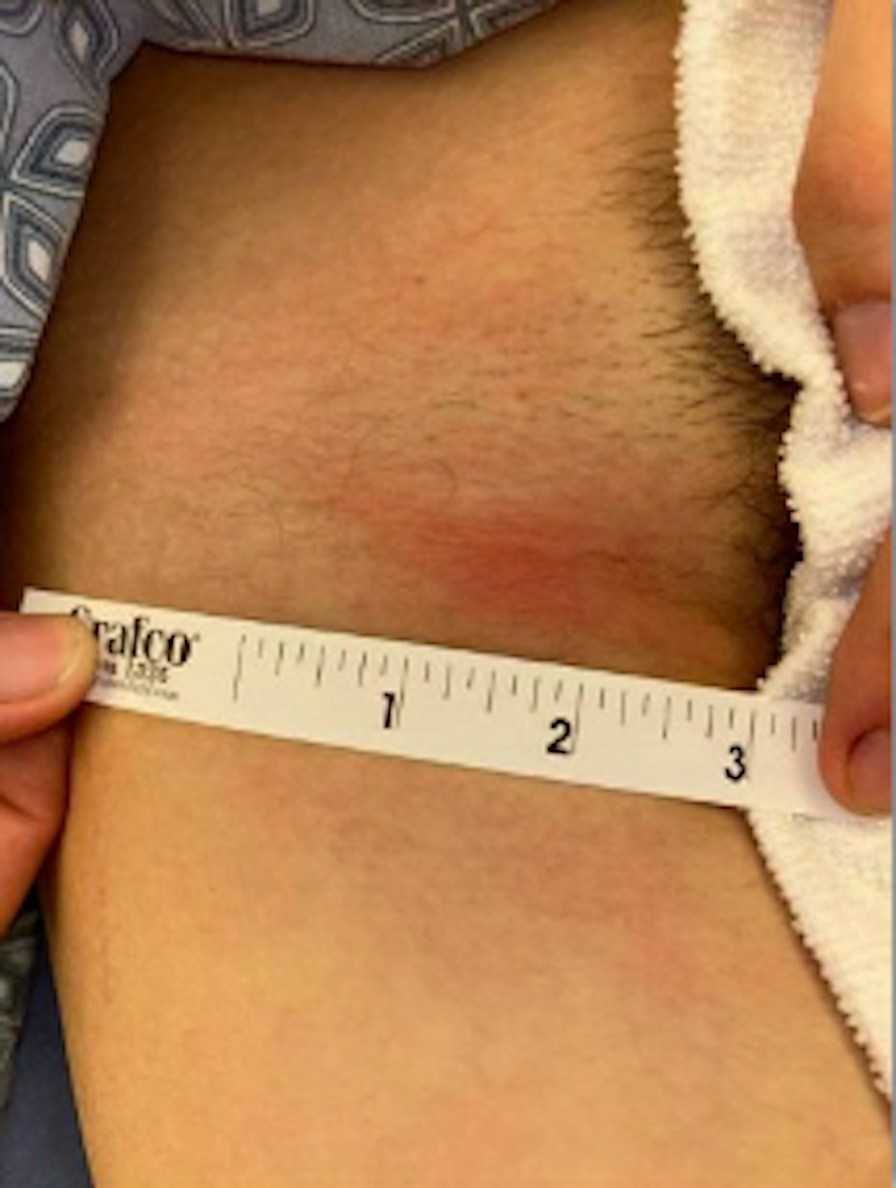
Right-sided inguinal lymphadenopathy.

On initial evaluation in the pediatric emergency department, the patient's vital signs were a temperature of 97.8°F, heart rate 80 beats per minute, respiratory rate of 16 breaths per minute, blood pressure 130/75, and oxygen saturation 98% on room air. He exhibited exquisitely tender bilateral inguinal swelling. The right inguinal region was warm to the touch with overlying erythema (Figure 1). There were no open lesions or pustules. Genitourinary examination revealed normal penile and testicular findings.

**Figure 2.**
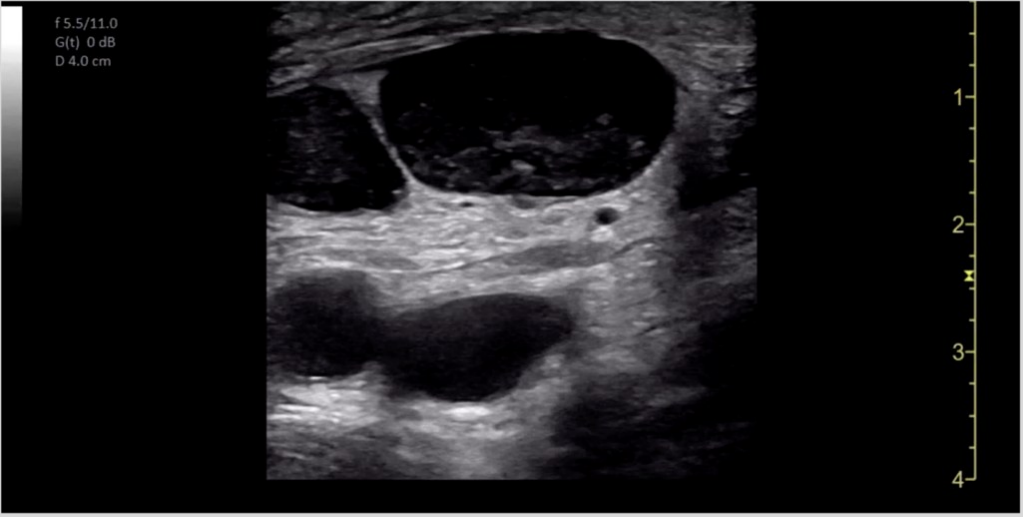
Point of care ultrasound (POCUS) of right inguinal region. Multiple enlarged lymph nodes superficial to the femoral artery and vein.

**Figure 3.**
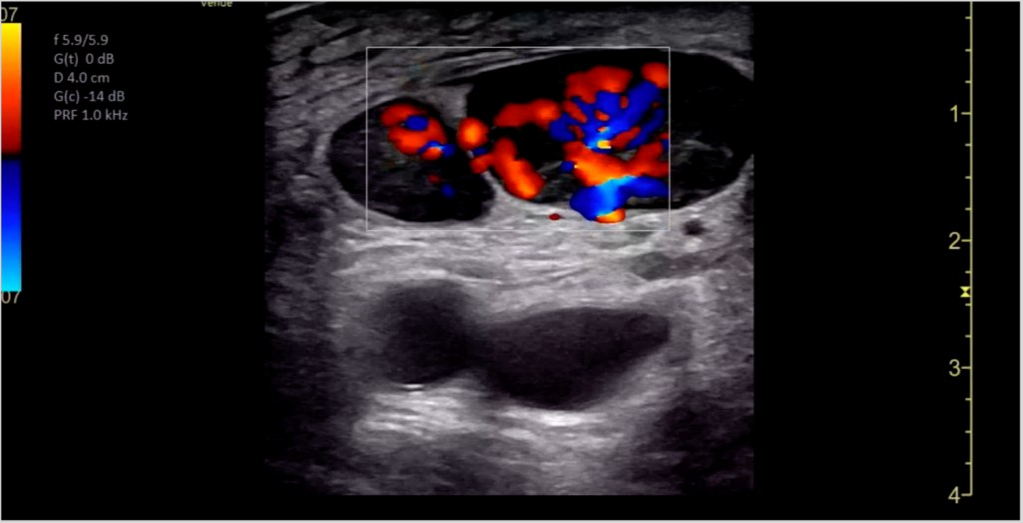
Point of care ultrasound (POCUS) with color Doppler. Diffuse internal vascularity is shown, previously described as “fire pattern.”

**Figure 4.**
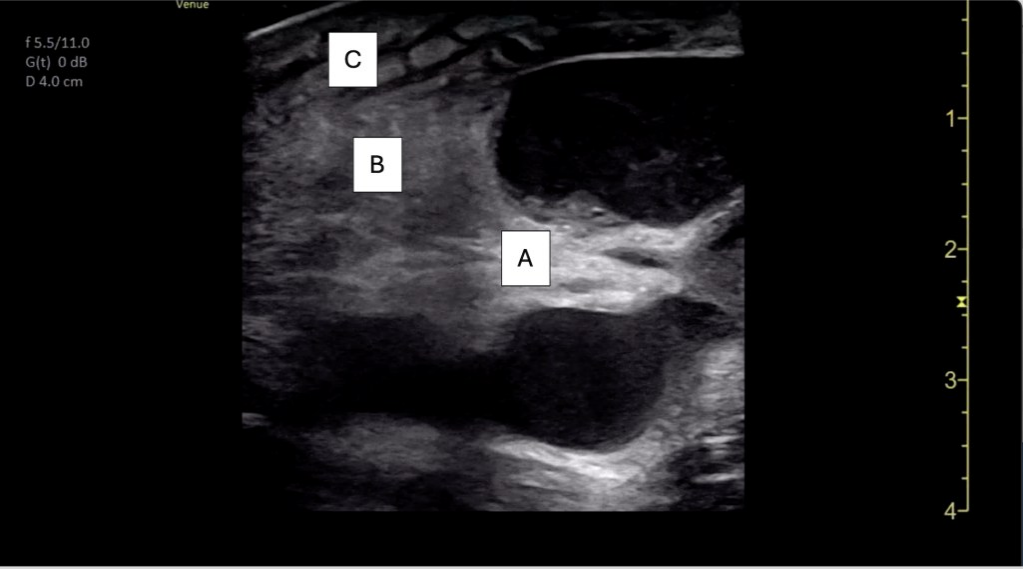
Point of care ultrasound (POCUS) image demonstrating posterior acoustic enhancement (A), soft tissue edema (B), and superficial cobblestoning (C).

POCUS of the skin and soft tissues was performed by a board-certified pediatric emergency medicine attending with emergency ultrasound fellowship training on a GE Venue machine (Waukesha, WI, USA), using a 4.2-13.0 MHz linear probe in a musculoskeletal preset. The POCUS examination revealed multiple bilateral hypoechoic ovoid structures with no identifiable hilum, the largest of which measured 1.9 cm in diameter (Figure 2). There was also markedly increased internal vascularity in a “fire-like” distribution within all the involved structures on color Doppler (Figure 3). Additionally, the POCUS examination demonstrated posterior acoustic enhancement, increased echogenicity of the surrounding soft tissue, and superficial cobblestoning (Figure 4). These POCUS findings, along with the notable absence of gut signature, internal heterogeneity, or free fluid, facilitated the identification of lymphadenopathy. The presence of posterior acoustic enhancement, increased echogenicity of surrounding soft tissues, and increased vascularity on color Doppler, in combination with physical examination findings of tenderness, warmth, and overlying erythema, were suggestive of infectious lymphadenitis with surrounding cellulitis.

Laboratory results revealed a white blood cell count of 17,100/µL (normal range: 4,500-9,200/µL) with 77.1% neutrophils, 1.1% immature granulocytes, 13.0% lymphocytes, 8.0% monocytes, 0.6% eosinophils, and 0.2% basophils, as well as C-reactive protein of 20.3 mg/L (normal: <10 mg/L), an erythrocyte sedimentation rate of 32 mm/hr (normal <15 mm/hr), and an unremarkable urinalysis, all of which supported the diagnosis of an infectious process. As a result, the infectious diseases team was consulted. The patient was initiated on doxycycline, providing coverage for both Bartonella spp. and methicillin-resistant *Staphylococcus aureus* (MRSA), as well as ceftriaxone, and was admitted to the hospital.

During hospitalization, additional laboratory testing were negative for human immunodeficiency virus, cytomegalovirus, and Treponema pallidum. The patient's Epstein-Barr Virus IgG was positive, consistent with prior infection. Blood flow cytometry for leukemia/lymphoma markers was negative. On hospital day 8, the patient's *Bartonella henselae* serology resulted with a titer of 1:256 (normal <1:64), supporting the diagnosis of CSD. The patient's symptoms slowly improved throughout his prolonged admission while receiving antibiotics (ceftriaxone was discontinued after 48 hours) and pain control. The patient received radiology-performed ultrasounds on hospital days 0, 3, 5, and 7. He was ultimately discharged on hospital day 9, as surgical intervention was deemed unnecessary, with a plan to complete a 10-day course of doxycycline as an outpatient.

**Figure 5.**
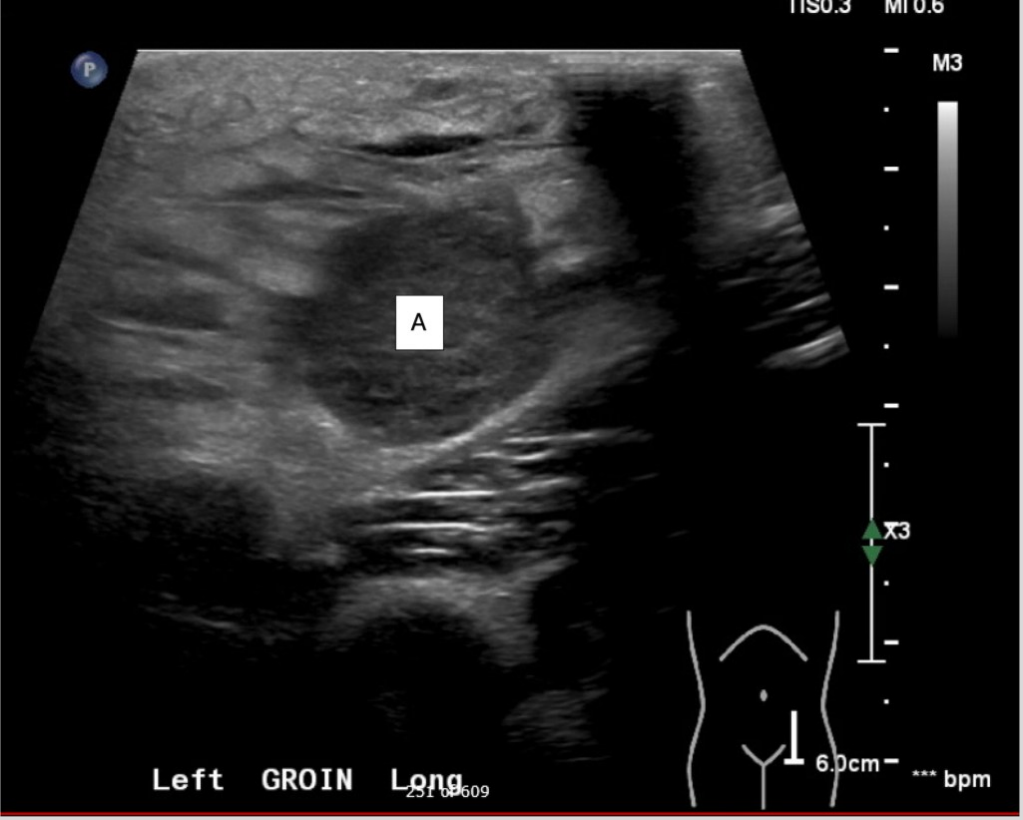
Radiology performed ultrasound of left groin during readmission demonstrating a heterogeneous peri-lymphatic fluid collection (A).

Three days after hospital discharge, the patient re-presented to the pediatric emergency department with worsening inguinal pain, edema, and erythema. A radiology-performed ultrasound was completed on the day of his readmission due to persistent symptoms and revealed a large peri-lymphatic fluid collection measuring 4.0x0.7x4.1 cm (Figure 5), raising concern for superimposed bacterial infection. He was readmitted and initiated on intravenous vancomycin and piperacillin-tazobactam. Pediatric surgery was consulted, and an incision and drainage procedure was performed. Wound cultures were obtained, and a lymph node excision was considered but deferred. Wound cultures remained negative for 48 hours, and the patient was discharged on hospital day 9 of his readmission, after transitioning to oral doxycycline, rifampin, and prednisone. Doxycycline was selected due to a favorable response during his 10-day admission.

At one-month follow-up with pediatric surgery and pediatric infectious disease, the patient's pain, swelling, and erythema had improved, though not completely resolved. Given the completion of two antibiotic courses for presumed *Bartonella* infection, no additional antibiotics were prescribed. However, the patient was instructed to continue a prolonged prednisone taper.

## Discussion

This case describes the use of POCUS during the initial evaluation of a teenager with acute bilateral inguinal swelling. POCUS enabled the emergency department team to promptly identify lymphadenopathy as the underlying cause of their inguinal soft tissue swelling. Using a combination of clinical findings and POCUS imaging, the team determined that infectious lymphadenitis was the most likely diagnosis. The POCUS findings in the emergency department, along with serial ultrasound examinations performed during hospitalization, guided clinical decision-making and eliminated the need for computed tomography or magnetic resonance imaging. This report also provides a brief review of CSD and its associated POCUS features.

Inguinal soft tissue swelling has an extensive differential diagnosis, which includes non-incarcerated hernia, incarcerated hernia, abscess, and malignancy. Specific causes of inguinal swelling have characteristic ultrasonographic features that were notably absent in this case: the presence of bowel, suggestive of a hernia; heterogeneity with internal debris, suggestive of an abscess; or a lack of compressibility, suggestive of a malignancy (Table 1) [1,2]. In this case, clinicians were able to rapidly identify lymphadenopathy as the underlying cause of soft tissue swelling. POCUS is an effective and widely accessible tool that should be considered an essential component of an emergency medicine physician's inventory when evaluating patients with soft tissue swelling.

### Ultrasound Evaluation of Lymphadenopathy

**Figure 6.**
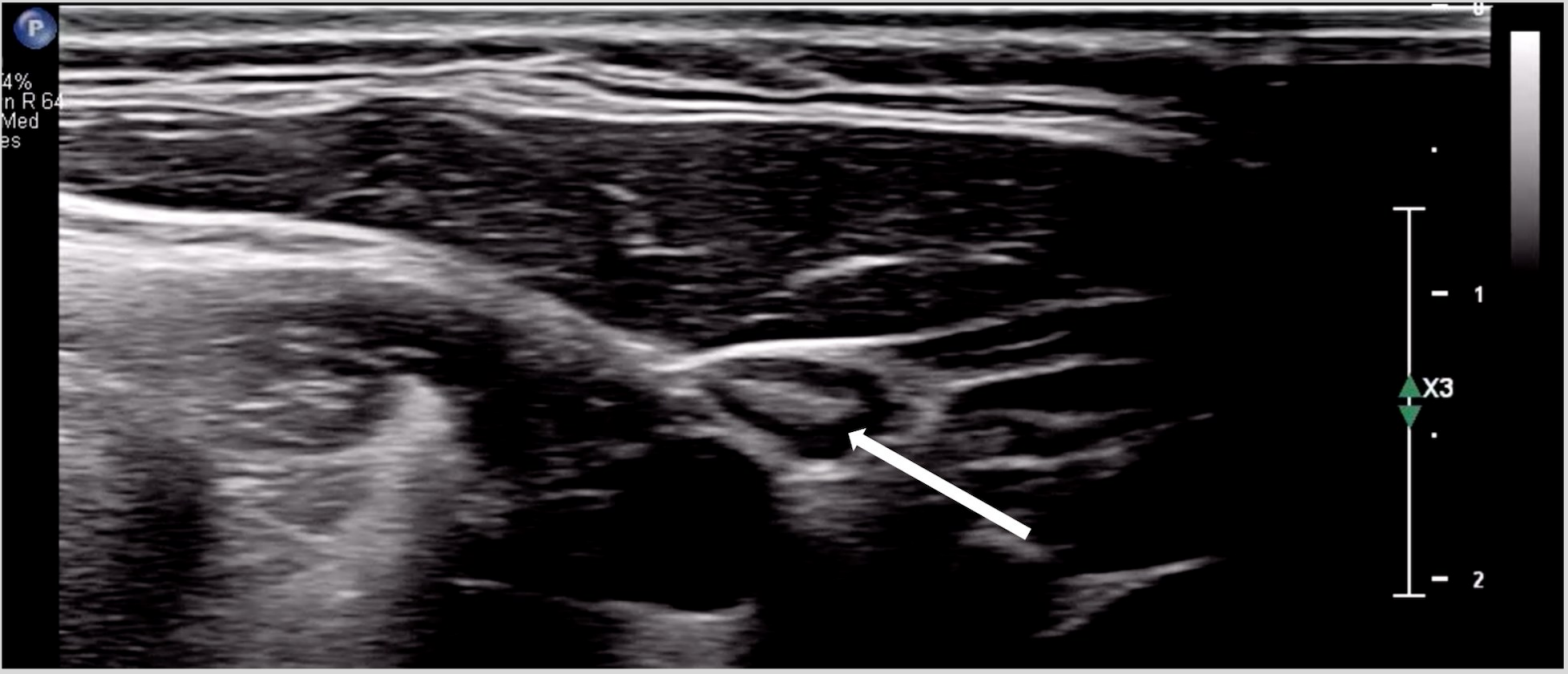
Point of care ultrasound (POCUS) of normal Lymph node (arrow indicating presence of hilum).

**Figure 7.**
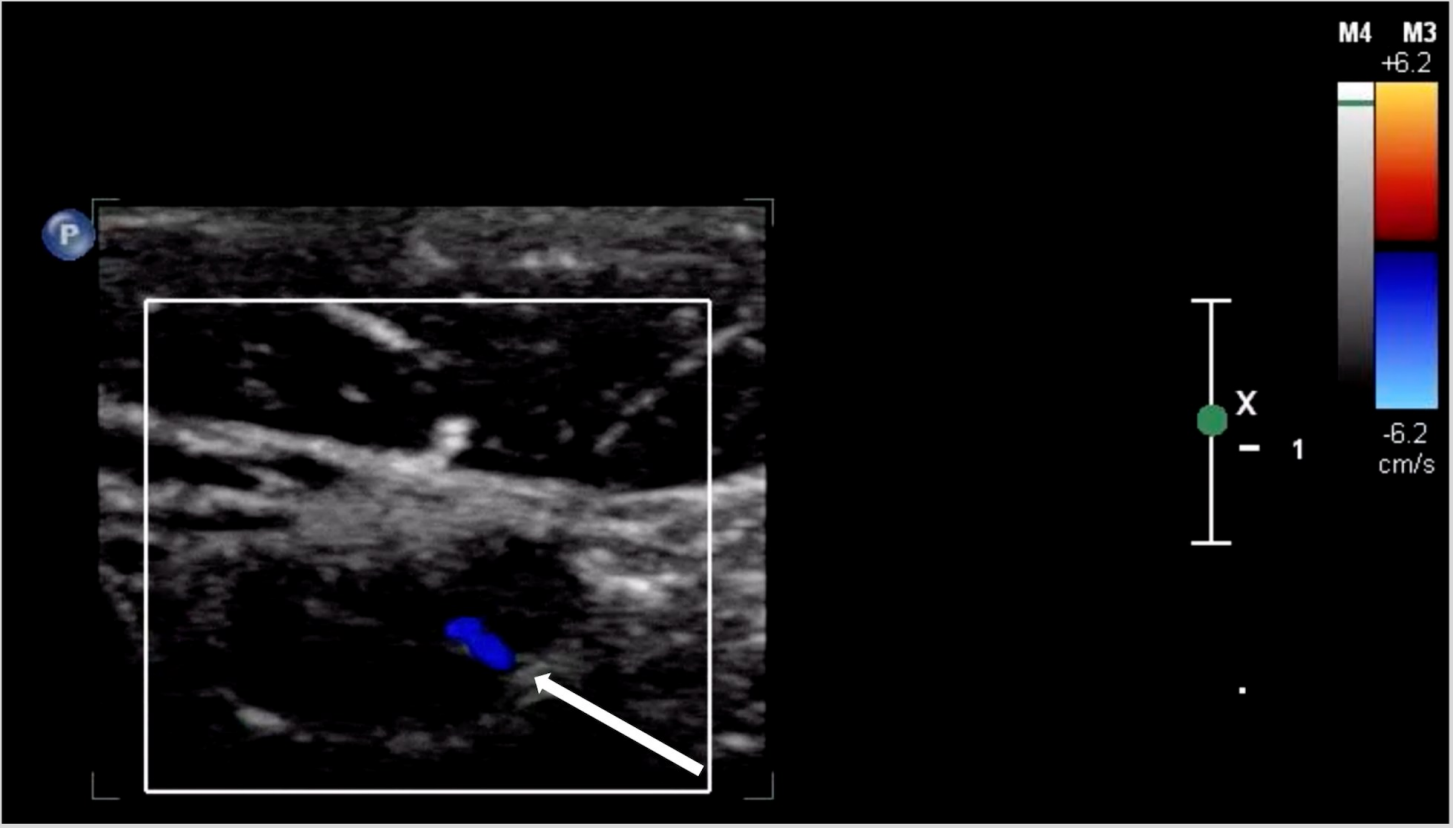
Point of care ultrasound (POCUS) with color Doppler applied to a normal lymph node (arrow indicating presence of hilum).

Ultrasound has been suggested as the first-line imaging modality for evaluating lymphadenopathy due to its rapid image acquisition and interpretation, lack of ionizing radiation, and low cost [7]. Normal lymph nodes are typically described as well-defined ovoid structures with hypoechoic parenchyma, an echogenic hilum, and hilar vascularity on Doppler imaging (Figures 6 and 7) [3]. Additional sonographic features, such as the presence of posterior acoustic enhancement, increased echogenicity of adjacent soft tissues, and specific color Doppler patterns, can aid in differentiating reactive, infectious, and malignant lymphadenopathy (Table 2) [3,4]. In this case, multiple hypoechoic ovoid structures without an identifiable hilum were visualized. Additionally, posterior acoustic enhancement, adjacent soft tissue edema, and an enhanced color Doppler signal were all present. The combination of hypoechoic echotexture, posterior acoustic enhancement, and the absence of an identifiable hilum likely represented liquefaction. Intranodal liquefaction is suggestive of necrosis and can help distinguish pathologic etiologies (Table 2) [3,4,8].

This case demonstrated a unique benefit of POCUS: images were acquired in real time in the emergency department and directly facilitated the initial management of the patient. While multiple radiology-performed ultrasounds were obtained during the patient's initial admission (hospital days 0, 3, 5, 7) and re-admission (hospital days 0, 4), only the initial POCUS examination was performed. Another potential benefit of POCUS is its capacity for iterative bedside examinations, which may provide valuable insight into the evolution of a disease process. Had a follow-up POCUS study been performed before the patient's initial discharge, a developing fluid collection may have been identified, potentially avoiding the need for readmission. This case highlights the need for expanded POCUS application in both acute care and inpatient settings.

### Cat Scratch Disease

A wide range of disease states and clinical symptoms can result from Bartonella infection, including CSD [9]. It commonly manifests as regional lymphadenopathy, typically preceded by an erythematous papule at the inoculation site [10]. The presentation of Bartonella-associated lymphadenitis in this case was uniquely challenging due to the isolation of adenopathy to the bilateral inguinal lymph nodes and the absence of an identifiable inoculation site. Bartonella most commonly affects cervical (26%) and axillary (46%) lymph nodes, while inguinal lymph nodes are less commonly affected (17%) [9]. Inguinal lymph nodes are the primary site of lymph drainage for the lower extremities, external genitalia, lower abdominal wall, perineum, and anus. Unilateral inguinal adenopathy has been previously described in cases of CSD, most commonly following inoculation of the lower extremities [11]. In our patient, who presented with bilateral inguinal adenopathy, an inoculation site was never definitively identified. However, to explain the bilateral distribution of lymphadenopathy, the site of inoculation likely would have been centrally located, for example, in the external genitalia, perineum, or lower abdominal wall. There has been one documented case of a three-year-old with bilateral inguinal lymphadenopathy attributed to CSD. However, this patient had disseminated disease with hepatic, splenic, and paravertebral lesions [12]. To our knowledge, bilateral inguinal adenopathy has not been reported as a primary manifestation of CSD.

### Ultrasound Findings in Cat Scratch Disease

Sonographic features of lymphadenopathy due to CSD have been previously described in the literature. García et al. reported the ultrasonographic findings in 47 patients diagnosed with CSD who underwent a radiology-performed skin and soft tissue ultrasound [4]. They noted multiple findings that were present in a majority of patients in the series, including the presence of three or more enlarged lymph nodes (91%), hypoechoic echotexture (100%), posterior acoustic enhancement (89%), and increased echogenicity of surrounding soft tissues (100%) [4]. The POCUS examination that was performed in our case demonstrated each of these characteristic findings. Additionally, all 21 cases in the García et al. series that were evaluated with color Doppler demonstrated “highly vascularized lymph nodes” with a loss of the typical vascular architecture. García et al. coined the term “fire pattern” to describe the diffuse color Doppler signal observed within the enlarged lymph nodes and postulated that this pattern was due to neovascularization associated with Bartonella infection [4]. We observed a similar distribution of Doppler signal in our case (Figure 3).

While radiology-performed ultrasonographic findings in CSD were previously reported in this single case series, our case is the first to describe the use of POCUS to facilitate the diagnosis of lymphadenitis ultimately attributed to CSD. It should be noted that although the described findings are associated with CSD, they are not specific. Lymph node hypervascularity has also been described in other infectious etiologies, such as tuberculosis, as well as malignancy. Additionally, the development of peri-lymphatic fluid collections, as seen in our patient, has been previously described as a sequela of CSD infections [13]. The fire pattern seen in this case may also provide a useful imaging clue that favors CSD over other etiologies [3,4]. In addition to its ability to identify lymph node manifestations of CSD, ultrasound has also been used to evaluate systemic manifestations of CSD. García et al. identified splenic and/or hepatic granulomas in 33% (10/30) of patients with CSD who underwent abdominal ultrasound [4].

Antimicrobial treatment is recommended for all patients with CSD-associated lymphadenitis to prevent the serious complications associated with dissemination, including hepatosplenomegaly, encephalopathy, and retinitis. A five-day course of azithromycin is the recommended first-line treatment, with combination therapy using azithromycin and rifampin for refractory cases [14]. In our case, infectious diseases recommended the use of doxycycline in place of azithromycin due to concerns about a possible superimposed gram-positive bacterial infection. Corticosteroids may be considered as adjunctive therapy for severe or persistent disease to aid in symptomatic management [14].

## Conclusion

This case demonstrated how POCUS can guide the evaluation and management of soft tissue swelling in the pediatric emergency department. Furthermore, the use of POCUS in the evaluation of lymphadenopathy can inform clinical decision-making, facilitate targeted diagnostic testing, and support timely specialty consultation. To our knowledge, this is the first published case report describing the use of POCUS to evaluate pediatric inguinal lymphadenitis ultimately attributed to *Bartonella henselae* infection.
